# The evolution of music and human social capability

**DOI:** 10.3389/fnins.2014.00292

**Published:** 2014-09-17

**Authors:** Jay Schulkin, Greta B. Raglan

**Affiliations:** ^1^Department of Neuroscience, Georgetown UniversityWashington, DC, USA; ^2^Department of Research, American College of Obstetricians and GynecologistsWashington, DC, USA

**Keywords:** music, evolution, social capability, cognitive capability, communication

## Abstract

Music is a core human experience and generative processes reflect cognitive capabilities. Music is often functional because it is something that can promote human well-being by facilitating human contact, human meaning, and human imagination of possibilities, tying it to our social instincts. Cognitive systems also underlie musical performance and sensibilities. Music is one of those things that we do spontaneously, reflecting brain machinery linked to communicative functions, enlarged and diversified across a broad array of human activities. Music cuts across diverse cognitive capabilities and resources, including numeracy, language, and space perception. In the same way, music intersects with cultural boundaries, facilitating our “social self” by linking our shared experiences and intentions. This paper focuses on the intersection between the neuroscience of music, and human social functioning to illustrate the importance of music to human behaviors.

## Background

Music is a fundamental part of our evolution; we probably sang before we spoke in syntactically guided sentences. Song is represented across animal worlds; birds and whales produce sounds, though not always melodic to our ears, but still rich in semantically communicative functions. Song is not surprisingly tied to a vast array of semiotics that pervade nature: calling attention to oneself, expanding oneself, selling oneself, deceiving others, reaching out to others and calling on others. The creative capability so inherent in music is a unique human trait.

Music is strongly linked to motivation and to human social contact. Only a portion of people may play music, but all can, and do, at least sing or hum a tune. Music is like breathing—all pervasive. Music is a core human experience and a generative process that reflects cognitive capabilities. It is intertwined with many basic human needs and is the result of thousands of years of neurobiological development. Music, as it has evolved in humankind, allows for unique expressions of social ties and the strengthening of relational connectedness.

Underlying the behavior of what we might call a basic proclivity to sing and to express music are appetitive urges, consummatory expression, drive and satisfaction (Dewey, 1925/1989). Music, like food ingestion, is rooted in biology. Appetitive expression is the buildup of need, and consummatory experiences are its release and reward. Appetitive and consummatory musical experiences are embedded in culturally rich symbols of meaning.

Music is linked to learning, and humans have a strong pedagogical predilection. Learning not only takes place in the development of direct musical skills, but in the connections between music and emotional experiences. Darwin understood both music and consideration of emotion to be human core capabilities. Emotional systems are forms of adaptation allowing us to, for instance, note danger through the immediate detection of facial expressions.

This essay examines the biological and cognitive context for musical expression. In addition, it looks at how the predilection for music among humans has helped to foster the social connectedness so unique and vital to our species, and how our cephalic capabilities underlie music. This paper suggests that the importance of music to our socialization and well-being as a species is reflected in the cognitive and neural connections underpinning it.

## The social functions of music

Music is often functional because it is something that can promote human well-being by facilitating human contact, human meaning, and human imagination of possibilities. We came quite easily, one might surmise, to the cephalic state of enjoying music for itself, its expanding melodic and harmonic features, its endless diverse expression of sound, moving through space, and within our power to self-generate it (Koelsch, 2010). On the voyage that conceptualized an important idea already circulating in Victorian culture—adaptation and natural selection—Darwin spent quite a bit of time studying the phenomenon of song. He was keen to understand song as a biological feature: “It is probable that the progenitors of man, either the males or females or both sexes before acquiring the power of expressing mutual love in articulate speech, endeavored to charm each other with musical notes and rhythm (Darwin, 1871/1874).” Darwin posited that song evolved with communicative capabilities, which extended for some species (e.g., song birds and humans) with great variation.

Musical sensibility is tied to our social instincts. Darwin noted as early as 1859 that social instincts, including song, are the prelude for much of what governs our social evolution (Darwin, 1859/1958).

Darwin and the ethologist Tinbergen understood that functions can change over time and be put to novel uses (Tinbergen, 1951). Musical expression requires a wide range of such functions: respiratory control, fine motor control, and other preadaptive features. This figures into song production, an evolution tied to speech and the diversification of our communicative competence.

Musical sensibility is surely just as fundamental to the human species as, for instance, language. From a simple adaptation there emerges lively expression in almost any culture. Music is indeed generative, structurally recursive, and knotted to grouping (Diderot, 1755/1964; Spencer, 1852).

Music is a binding factor in our social milieu; it is a feature with and about us, a universal still shrouded in endless mystery. How music came into being is, like most other features in our evolution, hard to pinpoint. Evolutionary evidence over a wide range of cultural groups reveals diversity of song and instrument, yet gaps and speculative considerations remain: some cultures sing a lot, some sing less, but most do sing and perhaps Neanderthals sang more than Sapiens (Mithen, 2006). Music is typically something shared, something social; we may sing in the shower or on a solitary walk (Whitehead, 1938/1967; Rousseau, 1966), but music is most of the time social, communicative, expressive, and oriented toward others.

Music cuts across diverse cognitive capabilities and resources, including numeracy, language, and spacial perception. In the same way, music intersects with cultural boundaries, facilitating our “social self” by linking our shared experiences and intentions. Perhaps one primordial influence is the social interaction of parental attachments, which are fundamental to gaining a foothold in the social milieu, learning, and surviving; music and song are conduits for forging links across barriers, for making contact with others, and for being indoctrinated with the social milieu.

Ian Cross (Cross and Morley, 2008; Cross, 2010), has pointed out the floating, fluid expression of music. There is little doubt that the fundamental link that music provides for us is about emotion and communicative expression, in which the prediction of events is tied to diverse appraisal systems expressed in music (Meyer, 1956; Sloboda, 1985/2000; Huron, 2006). Music is fundamental to our social roots (Cross, 2010). Coordinated rituals allow us to resonate with others in chorus (Brown, 2003), for which shared intentional movements and actions are bound to one another.

Culture-bound music is a shared resource that is tied to diverse actions, including sexual function (Darwin, 1872/1998). Music permeates the way in which we coordinate with one another in rhythmic patterns, reflecting self-generative cephalic expression (Temperley, 2001; Jackendoff and Lerdahl, 2006) tied to a rich sense of diverse musical semiotics and rhythms (Peirce, 1903-1912/1977; Myers, 1905). Music is embedded in the rhythmic patterns (Myers, 1905; Sacks, 2008; Cross, 2010) of all societies. Our repertoire of expression has incurred a crucial advantage: the ability to reach others and to communicate affectively laden messages.

The social communicative bonding of the wolf chorus is one example from nature that comes to mind (Brown et al., 2004); a great chorus of rhythmic sounds in a social setting. A common theme noted by many inquirers is the social synchrony of musical sensibility (Sloboda, 1985/2000; Temperley, 2001; Huron, 2006; Cross, 2010). The motor sense is tied directly to the sounds, synchrony and movement. Sometimes the actual motor side of singing is underappreciated (Brown, 2006). Neurotransmitters, which are vital for movement, are tethered to syntax and perhaps to sound production. The communicative social affective bonding is just that: affective. This draws us together and, as a social species, remains essential to us; a chorus of expression in being with others, that fundamental feature of our life and of our evolutionary ascent. Music is indeed, as Timothy Blanning noted, a grand “triumph” of the human condition, spanning across cultures to reach the greatest of heights in the pantheon of human expression, communication, and well-being. It is in everything (Cross, 1999; Huron, 2001).

We are a species bound by evolution and diverse forms of change, both symbolic and social. Language and music are as much a part of our evolutionary development as the tool making and the cognitive skills that we traditionally focus on when we think about evolution. As social animals, we are oriented toward sundry expressions of our con-specifics that root us in the social world (Humphrey, 1976), a world of acceptance and rejection, approach and avoidance, which features objects rich with significance and meaning (Marler, 1961, 2000). Music inherently procures the detection of intention and emotion, as well as whether to approach or avoid (Juslin and Sloboda, 2001; Juslin and Vastfjall, 2008).

Social behavior is a premium cognitive adaptation, reaching greater depths in humans than in any other species. The orientation of the human child, for example, to a physical domain of objects, can appear quite similar in the performance of some tasks to the chimpanzee or orangutan in the first few years of development (Herman et al., 2007). What becomes quite evident early on in ontogeny is the link to the vastness of the social world in which the human neonate is trying to gain a foothold for action (Tomasello and Carpenter, 2007). Music is social in nature; we inherently feel the social value of reaching others in music or by moving others in song across the broad social milieu.

## Social and musical contact and cortical expansion

**Figure 1:**
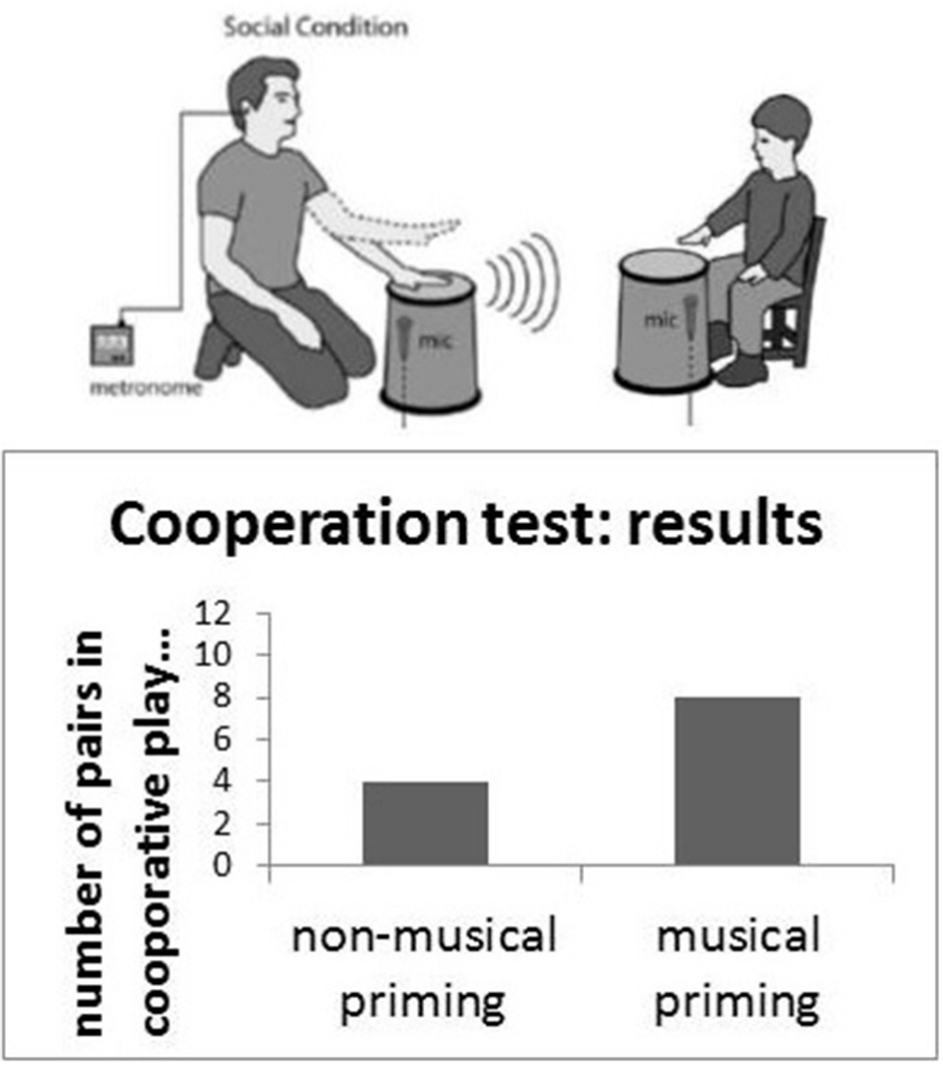
**Joint social action and music making; musical priming leads to an increase in the number of cooperative players (Kirschner and Tomasello, 2009, 2010)**.

Music is replete with social contact. In fact, its origins are in contact with others. Mothers making contact, calls to others, and rhythmic patterns with others in the social group are all ways of keeping track of others, staying in touch with others, or playing with others. Indeed, exposure to music in young children is known to promote prosocial behavior in children. Studies suggest joint singing or drumming, for instance, when controlling for diverse intellectual and personality factors, promotes prosocial behaviors (See Figure 1) (Kirschner and Tomasello, 2009, 2010).

**Figure 2:**
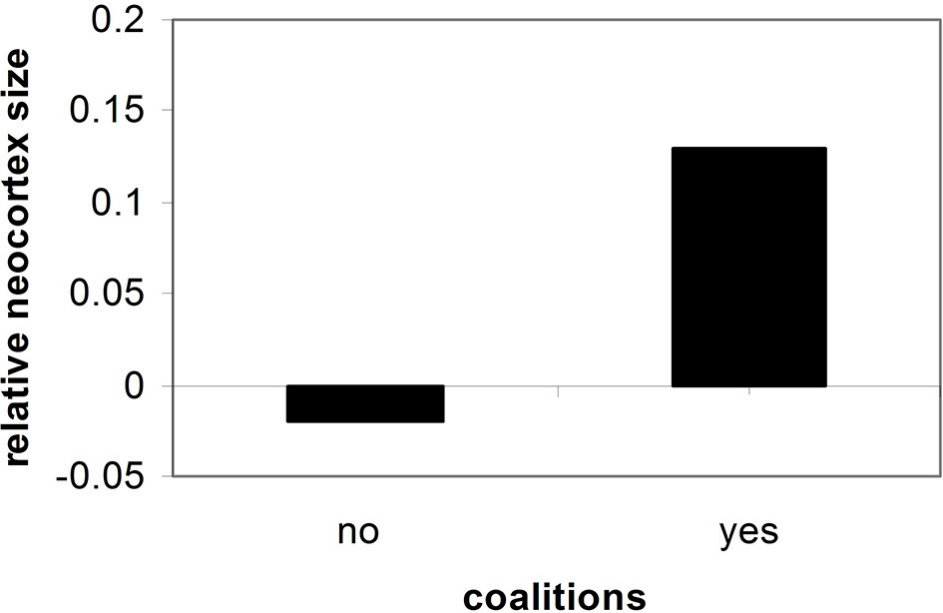
**Neocortex size and social cooperation (Dunbar and Shultz, 2007)**.

Importantly, the greater the degree of social contact and social organization experienced by a human, the greater the trend toward cortical expansion (See Figure 2) (Dunbar, 1996, 1998, 2003; Barton, 2006; Dunbar and Shultz, 2007).

In other words, group size and social contact is linked to neocortical expansion in hominids, as is longevity. The pressure of coming into contact with others, creating alliances, and tracking them no doubt required more cortical mass (Byrne and Corp, 2004; Cheney and Seyfarth, 2007).

Interesting correlations have been suggested between neocortical size and social cognitive skills (Byrne, 1995; Reader and Laland, 2002), and this extends to musical calls. It is the also the expansion of cephalic functions that underlie the tool use that make musical instrumentalism possible. An expanded cortical/motor system with diverse cognitive capacities is no doubt pivotal to our evolutionary ascent and to the musical instruments that we developed to facilitate social interaction (Reader and Laland, 2002; Barton, 2004; Schulkin, 2007). A broad based set of findings in non-primates has also linked social complexity to larger brain size (Byrne and Corp, 2004). Technology, including musical objects, is an extension of ourselves that expands what we explore (Heelan and Schulkin, 1998; Lakoff and Johnson, 1999), facilitating plasticity of expression and long term social bonds.

Two important pathways in the central nervous system underlie how we ascertain where an object is located and what it may be (Ungerleider and Mishkin, 1982). This segmentation is tied to sound and song (Rauschecker and Scott, 2009). Moreover, neurons in the premotor region, located within the frontal lobe are contained to a large extent within Brodmann's area 6. This region is importantly involved in the direction of action (Kakei et al., 2001; Passingham, 2008) including musical expression and auditory input (Zatorre et al., 2002; Rauschecker and Scott, 2009). Moreover, diverse regions of the temporal lobe have long been linked to social perception, eye gaze, and tracking the vector of visual systems of others, and would also underlie musical expression (Rolls and Treves, 1998; Emery, 2000).

## Williams syndrome, music and pro-social behaviors

Williams Syndrome is a genetic exaggerated pro-social orientation to the world, linked to the dysregulation of oxytocin that is tied to diverse forms of pro-social behaviors (Dai et al., 2012). As an example of the interrelatedness of musicality and sociality, individuals with Williams syndrome share a common genomic marker and other common features. Their full scale IQ is usually much lower than the general population, and they have great difficulty with numbers and math. Their spatial capability is quite poor, although their linguistic capability is often good (Landau and Hoffman, 2005). Interestingly, motion processing in individuals with Williams syndrome is not perfect but remains fairly good (Reiss et al., 2005), suggesting that the ventral stream linked to motion and agency is operative. But the hypersociality associated with Williams syndrome is its most marked feature.

Often described as having “cocktail party” personalities, individuals with Williams syndrome are exceedingly cheerful, associate easily with strangers, and hyper-focus on eye contact when socially engaged. Thus, while expressing deficits in some intellectual capabilities, individuals with Williams syndrome nevertheless have intact and highly evolved human expression, including a greater liking of music, and may have much greater than average expression of perfect pitch (See Table 1).

**Table 1:** **Various features of Autistic and Williams patients**.

	**Autism**	**Williams**
Sociability	Low	High
Musical engagement	Typically Low	High
Empathy	Low	High
Cerebral volume	Normal	Small
Paleocerebellar volume	Normal	Small
Neocerebellar volume	Small	Large

*Source: (Levitin, 2005)*.

Children with Williams syndrome show a general decrease in brain volume (Galaburda et al., 2001). Regions of the temporal lobe are, however, actually greater in Williams syndrome than in controls (Reiss et al., 2005), while the amygdala is decreased (Galaburda et al., 2001). The amygdala of such children seems to be more reactive than controls to diverse social events (Haas et al., 2009).

Preserved musical sensibility in individuals with Williams syndrome is remarkable. Several studies have shown a greater liking of music in these individuals than age-matched controls (Don et al., 1999; Levitin et al., 2004). Williams patients more readily engage in music than controls, while autistic patients show decreased perception of emotion in music (Levitin and Bellugi, 2006; Bhatara et al., 2010). The hyper-social feature overlaps with a tendency toward hyper-musical engagement (Huron, 2001; Levitin et al., 2004). This engagement includes increased frequency in looking for music, playing music, and expressing emotional responses to music. A sensibility for and a sensitivity to sound seem to be features of these individuals (Levitin and Bellugi, 2006).

The temporal activation to music in controls vs. Williams syndrome individuals demonstrates activation of the temporal gyrus and Heschl's gyrus, while also showing a more diverse and diffuse activation that includes the amygdala and cerebellum (Levitin et al., 2003). Moreover, oxytocin, a prosocial facilitating peptide, may be elevated in Williams syndrome, and like dopamine, may be elevated when listening to music.

Individuals with Williams syndrome have also been reported to have an expanded activation of the visual cortex. In a study using functional magnetic resonance imaging (fMRI) to measure brain activity, individuals with Williams syndrome displayed greater visual cortex activation in response to music (Thompson et al., 1997). In addition, they showed diminished responses to anxiety associated with music (Dykins et al., 2005).

## Cognitive/emotional context

Music is an affectively opulent activity, whether it is being created or consumed. Moreover, music is rich in information processing as we work to appreciate the subtleties of beat, form, melody, and harmony. The affective and intellectual complexity of the musical experience speaks to the underlying neurological structures in place to ensure human appreciation for, and creation of, novel music.

**Figure 3:**
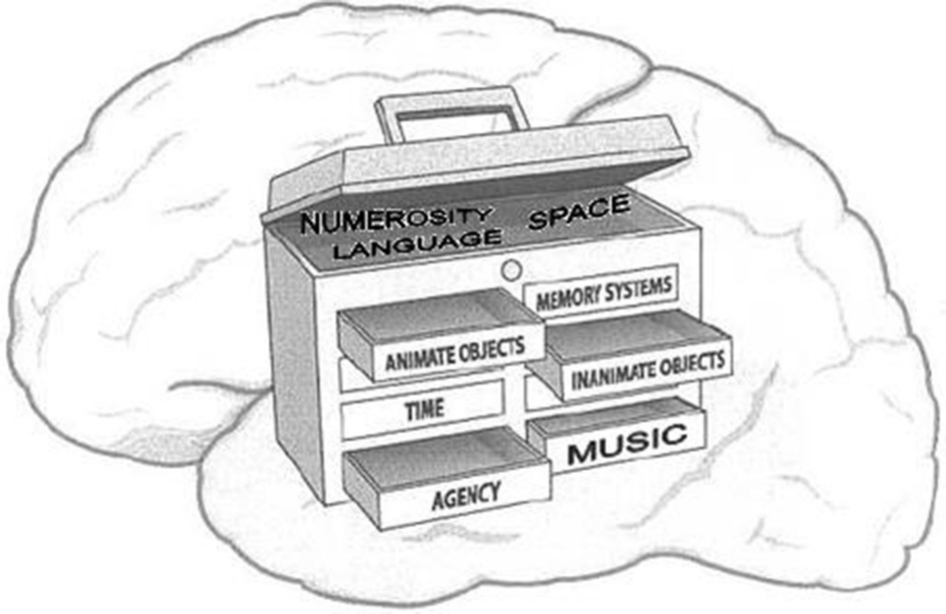
**A depiction of a toolbox as a metaphor for diverse cephalic capacities (Schulkin, 2009)**.

We come prepared with a cognitive toolbox that allows us to readily recognize animate objects, to sense time and space, to use language, and to discern agency in others (See Figure 3).

Gibson (1979) suggested that there is direct cephalic access to environmental sources of information and practices in the organization of action. Thus, some questions are: what are the conditions for adaptation and what are the factors in the environment that allow for readily available resources? This view of cognitive resources is linked to the ecological/social milieu, to what is available, what is dependable, what is utilizable, as well as the ability to use and unload information into environments that expand, enable, and bolster memory function as core cognitive events (Donald, 2001; Hatten, 2004; Clarke, 2008).

Context helps to facilitate performance, musical and otherwise. Our ways of hearing and responding to music are steeped in the direct ecological exposure to and expectations about sound and meaning, as well as music and context (Clarke and Cook, 2004). It is this sense of grounding that makes features stand out so easily in music and enables the mutualism between the perception, action, and external events that are quite palpable in music sensibilities (Clarke and Cook, 2004). The events are always relative to a framework of understanding—a social context rich in practice, style and history.

As well as providing a basis for understanding musical expression, context also affords an anchor with which to develop memories and future expectancies about music (Donald, 1991; Noe, 2004). The expansion of memory facilitates the wide array of what we do, including music. The emphasis is on action and perception knotted together and coupled with musical events.

The study of music emphasizes its independence from language while tying it, like all of our cognitive functions, to a diverse set of cognitive capabilities. Moreover, common forms of mental representations underlie action and perception in musical performance and musical sensibility (Deutsch, 1999; Pfordresher, 2006). Music is not only linked to cognitive actions, but also to emotional responsivity and memory formation.

## Adaptation, evolution, and music

**Figure 4:**
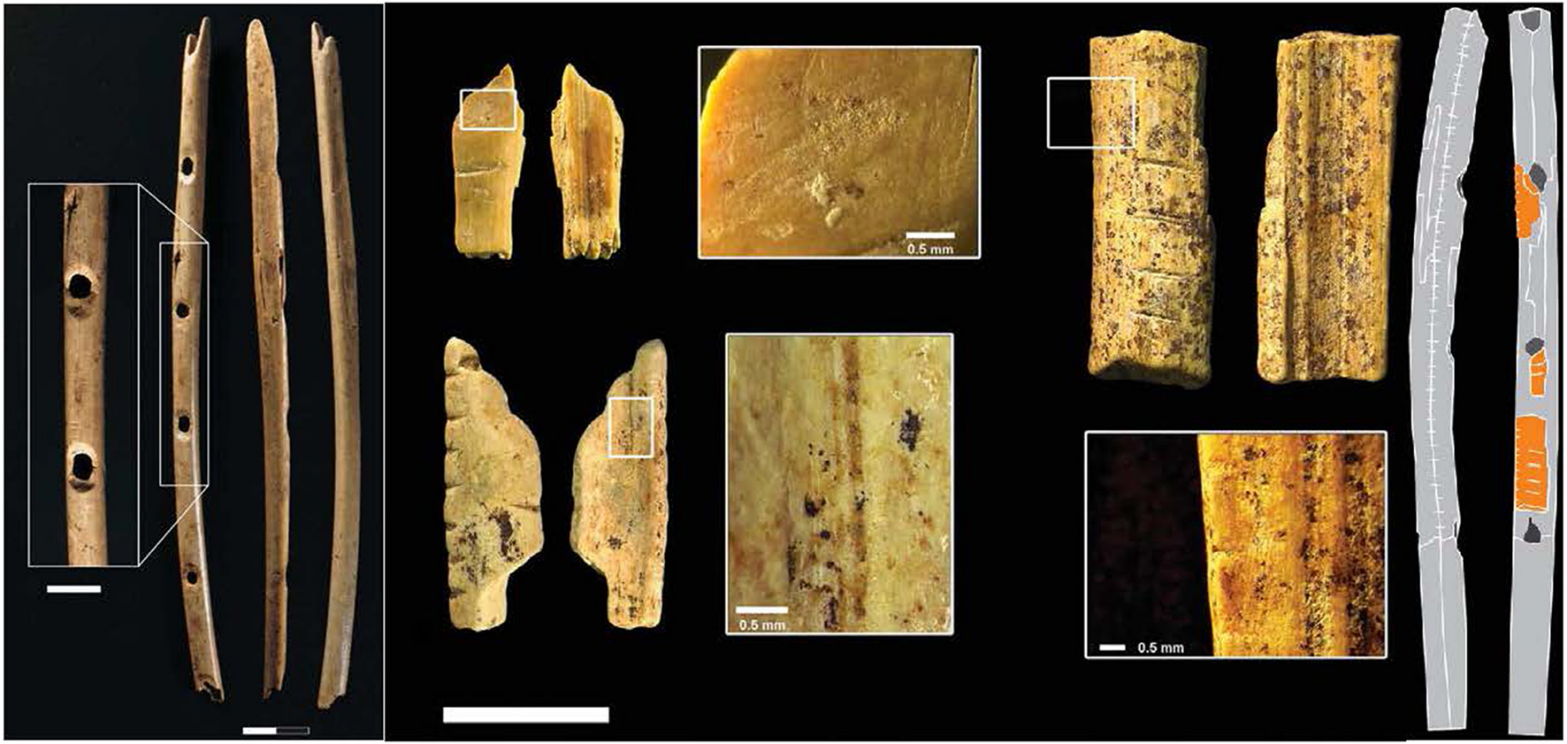
**Bone and ivory flute fragments from the Hohle Fels and Vogelherd caves in southwestern Germany (Conard et al., 2009)**.

From simple percusives to facile musical instruments, the tools of music represent a small leap for humankind. Diverse forms of art, tools, and probably music emerged in early *Homo sapiens*, and are evident in remains that date back at least 40,000 years (See Figure 4) (Mellars, 1996, 2004).

One cognitive adaptation is the capacity for the basic discernment of inanimate objects from animate objects. We represent animate objects, often giving them divine-like status, which infuses them with specific and transcendental meaning.

**Figure 5:**
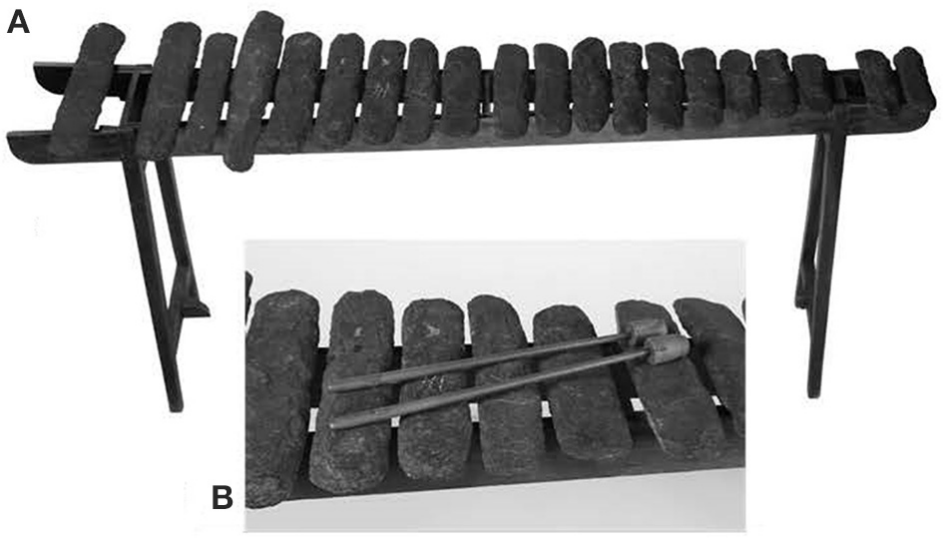
**Flint sound tool, known as a lithophone, from the Victorian Era (Blake and Cross, 2008)**.

Musical instruments ultimately derive from this expanded cognitive approach to objects. A key artifact is something that is sometimes called a “sound tool” or “lithophone.” The oldest date back to some 40,000 years ago from sites in Europe, Asia, and Africa (Blake and Cross, 2008). Sound tools are simple stones that resonate when struck, as shown in Figure 5.

While song is the earliest form of music, the cognitive and motor capabilities necessary for the invention of musical instruments are embedded in evolutionary cognitive development over time (Cross and Morley, 2008; Cross, 2009). After all, making objects, musical, and otherwise is a cephalic extension of the world beyond ourselves (Donald, 2001).

Darwin was prepared to believe that musical expression, as a particular universal human expression, is a feature of natural selection, linked to communicative function and sexual selection (Darwin, 1871/1874). Perhaps it is tentatively tied in origins to basic functions, but surely one wants to be respectful of these simple origins without being reduced to them.

Evolutionary trends are not necessarily unidirectional, as Darwin had suggested and had penned in one of his rather unaesthetic drawings. Evolutionary trends may be more like jumps and starts, punctuated by sudden changes (Gould and Eldridge, 1977; Foley, 1996; Wood, 2000).

One view of evolution is the hypothesis that language and speech emerged between 50,000 and 100,000 years ago (Lieberman and McCarthy, 2007), and artistic representation can be traced back to 30,000–40,000 years ago (Mellars, 1996).

Music, while frequently considered an art, captures the sciences in its generative process, and draws on human expectations. The cognitive architecture, the generative processes, the diverse variation and embodiment of human meaning within almost all spheres of human expression, are rich fields of discovery for both the arts and the sciences (Dewey, 1896; Meyer, 1967; Premack, 1990; Schulkin, 2009). This development of art and music was an important evolutionary step in forming the communicative scaffolding for social interactions that have become so crucial or our species.

Art, like science, is embedded in discovery, testing, experimentation, and expansion through technique. There is no divide between the scientific and artistic. They intersect quite readily and naturally as they expand the human experience.

## Action, music, and the brain

Given the key role that music plays in our social world, it is perhaps not surprising that music activates broad neurological systems, and that cognitive structures are in place for receiving, understanding, and producing music. Important biologically derived cognitive systems are not divorced from action or perception, but are endemic to them (Peirce, 1878; Barton, 2004; Schulkin, 2007).

Lakoff and Johnson (1999) depict relationships between perception and action, which underlie all of music, with thinking, perceiving, communicating, imagining, etc. Music is an action, but can also permeate our imagination, whether it is heard by someone, or simply imprints on neural systems. Music plays inside our heads, and as we shall see, common neural circuits underlie the action of playing and hearing music, as well as imagining music in reverberation (See Table 2) (Myers, 1905).

**Table 2:** **Relationships that underlie all aspects of musical experience**.

Thinking (music) as perceiving
Imagining (music) as moving
Knowing (music) as seeing and responding
Attempting insight (through music) as searching
Representing (music) as doing
Becoming- aware (of music) as noticing
Communicating (music) as showing
Knowing (music) from a “perspective”
Listening as detecting, knowing
Lakoff and Johnson, 1999

Music is fundamental to humans as a species. Most of the expectations we have may not be explicit, since the vast array of the cognitive systems are not conscious (Rozin, 1976); imagine playing an instrument while being explicitly conscious of all that we have to do. Impossible (Sloboda, 2000, 2005)! Cognitive systems are vastly unconscious and underlie action as well as music. The inferences, expectations, and prediction of auditory events are not particularly part of our awareness, and certainly the mechanisms are not (Helmholtz, 1873; Temperley, 2001).

**Figure 6:**
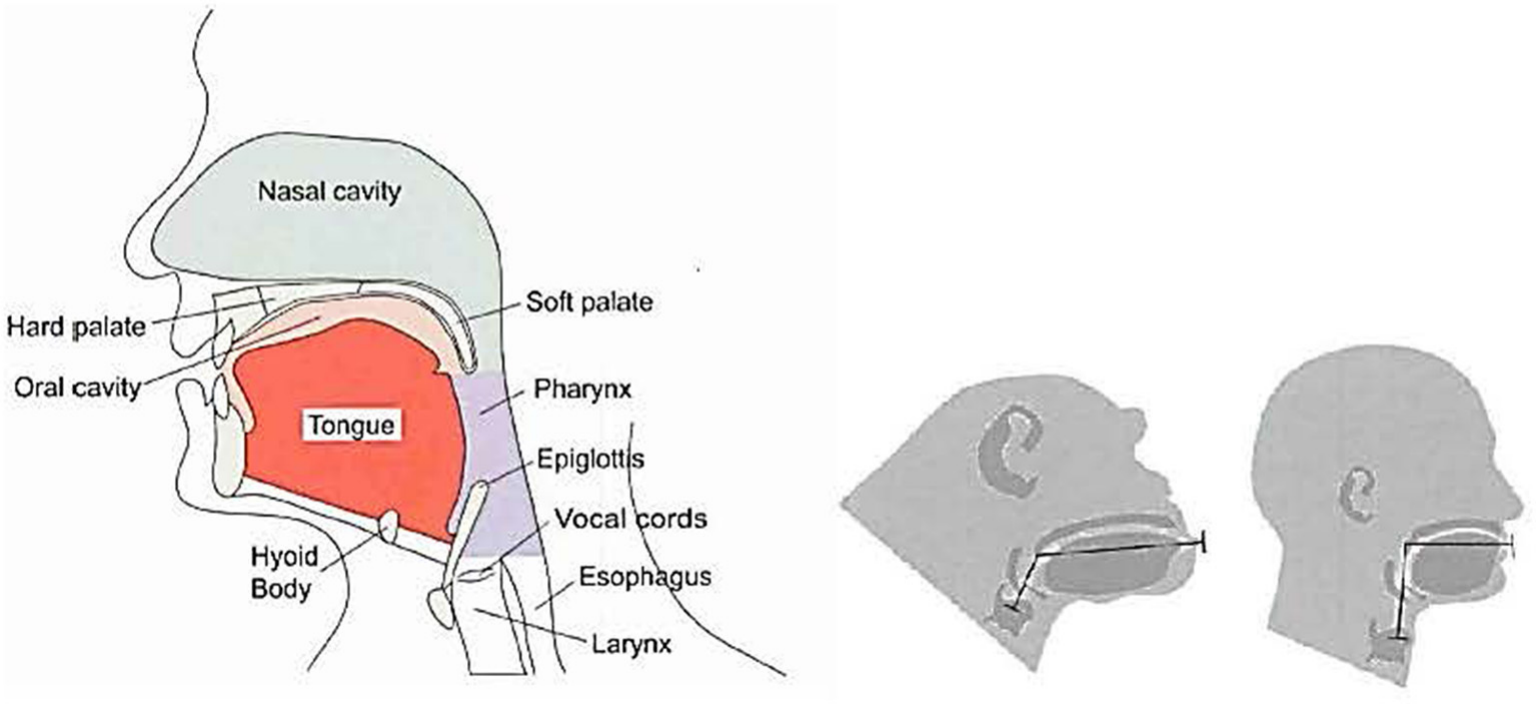
**Key features in the vocal capability of a chimpanzee (center) vs. a human (left, right) (Lieberman and McCarthy, 2007)**.

A core anatomy that includes a larynx (Lieberman, 1984) tied to systems which orchestrate movement featuring statistically related acoustical harmonics and periodicity is responsible for song production. These are bound to preferences for ratios and intervals between sounds via the modulation of the larynx (Ross et al., 2007). The expansion of the larynx, along with the development of cognitive/motor capability and “recursive thinking,” underlies speech, song, music, and other social communicative cephalic expressions (Corballis, 2007). These features figure in key adaptive responses that underlie our social capability (See Figure 6).

Access to pre-adaptive systems makes a difference in diversity of expression (Rozin, 1998; Fitch, 2006; Lieberman and McCarthy, 2007). As one investigator put it: “The larynx is a source of acoustic energy, not unlike the reed in a wind instrument (Lieberman, 1984, p. 317).” Communicative capabilities are endlessly opportunistic in the exploitation of existing resources with diverse and expanding uses.

More generally, auditory perceptual systems code and structure events for music within contexts of semiotic systems, which then further expand our capabilities for song. The evolving motor cortex, united with cognition and perception, underpin the production and appreciation of song (Lieberman, 1984, 2002). Music as we know it could not have existed without cognition or the motor skills to create musical sounds.

Diverse forms of cognitive systems reflect brain evolution (Rozin, 1976, 1998) with musical sensibility distributed across a wide array of neural sites, something that Leonard Meyer, an early exponent of a cognitive/ biological perspective, appreciated.

## Imagining and music

Positron Emission Tomography (PET) measures blood flow and is used as a marker of brain activation. In studies that used neuromagnetic methods to measure cortical activity, the primary motor cortex is active both when subjects observed simple movements and when the subjects performed them (Hari et al., 1998). Of course the motor cortex is activated in a wide array of human cognitive/motor activities. Importantly, motor imagery is replete with cognitive structure and is reflected in the activation of neural circuitry (Rizzolatti and Arbib, 1998), and so auditory imagery is reflected in different regions of the brain, including anticipatory musical imagery (Rauschecker and Scott, 2009).

In another study focusing specifically on sensory events in a fMRI scanner, subjects were presented with spoken words via headphones. Then, in a second experiment the same individuals were asked to identify the words with silent lip-reading (Calvert et al., 1997). Not surprisingly, many of the same cortical regions were activated. In other words, hearing sounds is like imagining them.

**Figure 7:**
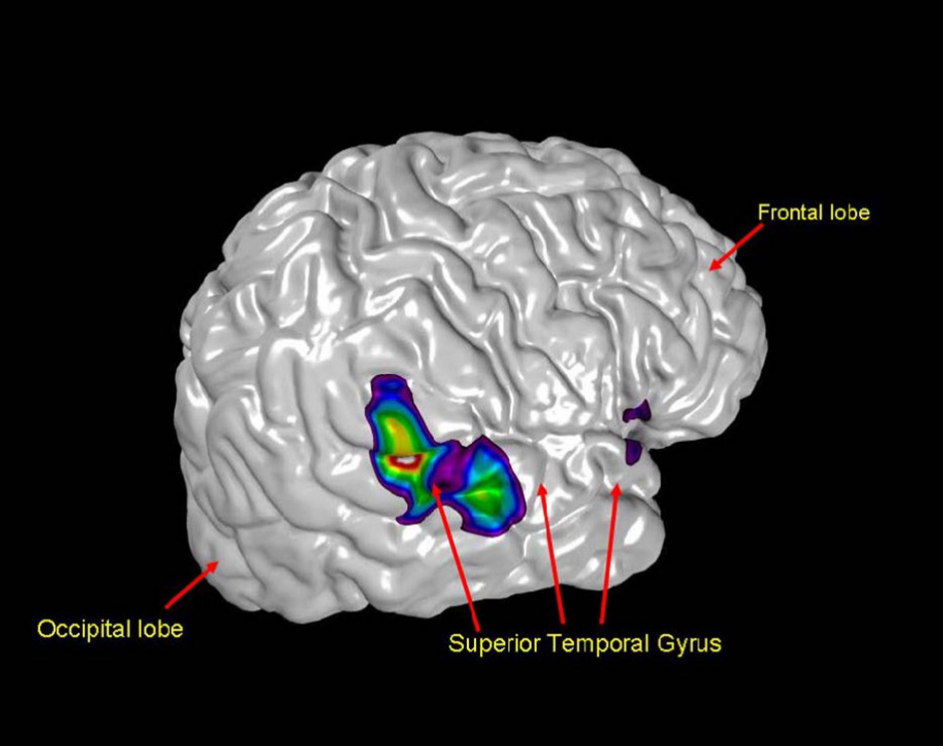
**A neuroimaging scan revealing that even in silence the auditory cortex, pictured here in the posterior portion of the right superior temporal gyrus, is activated (Zatorre and Halpern, 2005)**.

Not surprisingly, hearing music activates many of the regions linked to auditory perception. However, regions of the auditory cortex are also activated when subjects are asked to imagine music or other auditory stimuli (Figure 7) (Zatorre et al., 2002; Zatorre and Halpern, 2005).

Thus, despite the difficulty of knowing what people are actually imagining, one can dissociate hearing something from seeing it through diverse regions of the brain. Perhaps one is now in a better position to understand the genius of Beethoven; deaf for years, he must have heard music imaginatively to compose the way he did. Think of the cognitive complexity, the richness of the later parts of Beethoven's life. In fact, we now know that musical hallucinations are often a feature of acquired deafness such as Beethoven's (Zatorre et al., 2002). In addition, the links between audition and premotor cortex functioning mean that there is mutual activation, even in the absence of one or the other sensation (Baumann et al., 2007; Jäncke et al., 2012).

Of course, it also makes it somewhat easier to understand that the same “music to one's ears” may not be heard by one's neighbor. Beethoven is one thing, the rest of us quite another. Yet, the recruitment of cortical regions is generic.

## Dopamine, neural circuits, and music

Dopamine is a central organizer of drives and rewards and is tied to music sensibilities imagined, acted, and expected (Zatorre and Salimpoor, 2013). The regulation of dopamine is, for behavior, a fundamental event. It is an ancient molecule dating back millions of years in evolutionary history and plays a critical role in the motor control of the nervous systems of all vertebrates.

Dopamine levels are linked to diverse motivated behaviors (Kelley, 1999). These links have led a number of investigators to connect dopamine to reward. However, dopamine neurons are activated under a number of conditions, including duress or excitement. The pain of performance rituals through rehearsal and the expected excitement of the musical experience in context with others, for instance, activate dopamine.

Dopamine underlies the feeling of effort (practice, practice, practice, and yet more practice), and the rational prioritizing of our goals. Dopamine is active, we suggest, under both positive and negative conditions. For instance, either when one approaches something wanted or needed or when avoiding something aversive, dopamine is involved. In addition, dopamine is uniquely activated by the musical experience (Salimpoor et al., 2011).

Diverse cognitive resources are embedded in musical performance to reach out to the audience: the social milieu. Of course, musicians have to balance a sense of reward with the pain that they might be experiencing. They have to withstand short-term discomfort and set their sights on anticipatory, longer-term satisfaction (Sterling, 2004).

Music is action oriented, whether literally in the movement or the virtuosity of a Liszt, or in the controlled building up to a crescendo and release as in “The Lark Ascending” by the 20th century composer Vaughan Williams (Kennedy, 1964). Action permeates music and dopamine underlies the action of thought and the diverse cognitive systems that orchestrate the embodied expression of music.

An interesting set of studies on dopamine neurons in the brains of macaques has suggested that one function of this neurotransmitter is the prediction of rewarding events (such as hearing music) (Zatorre, 2001); dopamine neurons tend to fire more in anticipation of rewarding events.

**Figure 8:**
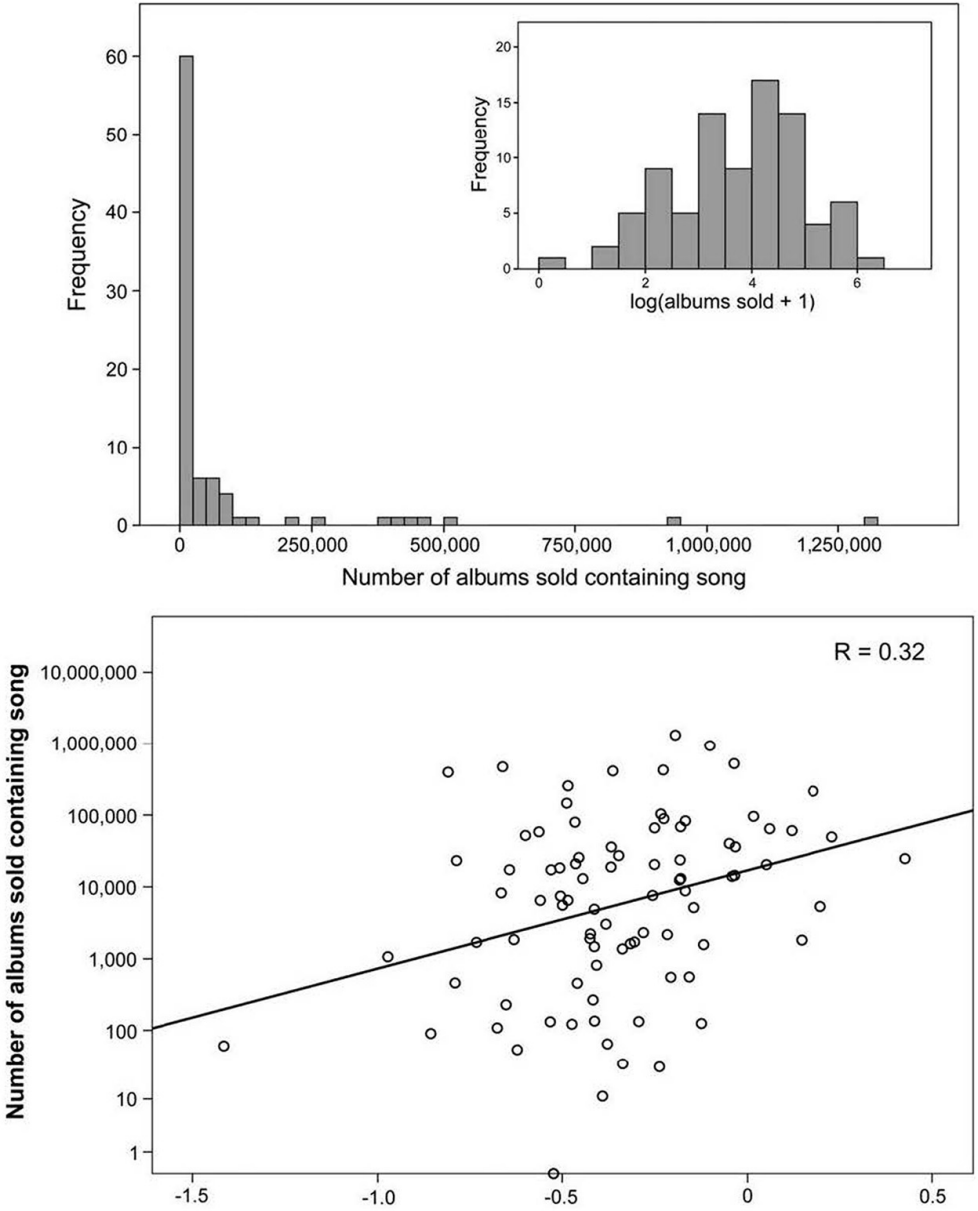
**The number of albums sold with the correlating activation of the nucleus accumbens (Berns and Moore, 2012)**.

Interestingly, using fMRI as a measure of brain activity shows that the activation of the nucleus accumbens is a predictive factor in the ratings of music (Blood et al., 1999; Menon and Levitin, 2005; Zatorre and Salimpoor, 2013). In fact, greater activation has been linked to a higher likelihood of purchasing of popular music in the United States (see Figure 8; Berns and Moore, 2012). Dopamine is not simply a neurotransmitter underlying the brain mechanisms linked to reward. It is much more complex and context-specific, such that even when dopamine is blocked, animals can still “like” things (e.g., sucrose). Indeed, dopamine is more tightly linked to the motivational component of pleasure-related events, and can be separated from the predictive reward components, while some of the endorphins are linked to the ingestion of a reward.

## Musical experience and changing the brain

Evidence suggests that the brains of musicians and non-musicians are different (Münte et al., 2002; Jäncke, 2009). Music shapes the cephalic encoding of information processing across different levels of the brain, from brainstem to cortex (Satoh et al., 2001; Patel, 2007; Wong et al., 2007; Cohen et al., 2011). Indeed, early musical training affects children's linguistic expression, and perhaps they are more sensitive in neonatal development (Marin, 2009) and on multisensory functioning (Stegemoller et al., 2008). Moreover, musical training enhances auditory capability more generally by impacting cortical and subcortical regions (Tramo et al., 2002; Kraus and Chandrasekaran, 2010).

In one study, for instance, gray matter differed between the two groups in the motor, visual and auditory cortex (Gaser and Schlaug, 2003). This may be due to enhanced neural connectivity. One set of studies suggests that in the corpus callosum, the main commissures between the two cortical hemispheres are greater in musicians vs. non-musicians (Schlaug et al., 1995).

In addition, intra-temporal lobe connectivity is increased in musicians with absolute pitch (Loui et al., 2010; Jäncke et al., 2012). This means that hearing tones more acutely is associated with greater inter-temporal neural connectivity. Based on this information, it would appear that several regions of the brain are altered and/or expanded by the hours of musical practice typically exercised by musicians. In fact, the actual extent of regular musical rehearsal practice is positively correlated to the degree of neural connectivity. The auditory cortex and the auditory systems more generally are intimately tied to music and hearing, including speech and song (Zatorre et al., 2002).

**Figure 9:**
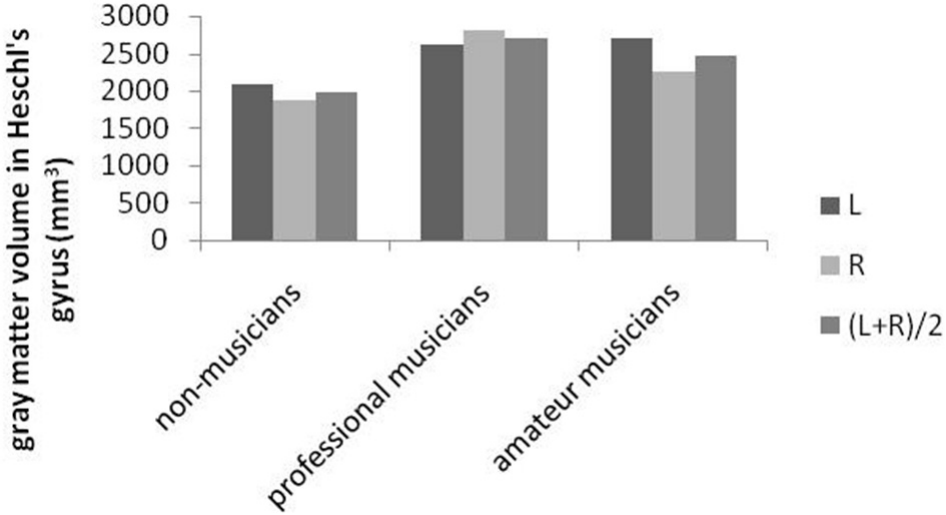
**Variations in Heschl's gyrus in the left and right hemispheres across three different groups (Schneider et al., 2002)**.

Music is richly organized into lexical networks of musical meaning (Peretz et al., 2009). One suggestion is that the left hemisphere, especially the superior region and surface of the temporal lobe (Heschl's gyrus), is tied to speech, and the right side is tied more to tone (Peretz et al., 2009). In two studies, for instance, the gray matter in the right cortical area was significantly greater in musicians (Keenan et al., 2001; Schneider et al., 2002; Zatorre and Halpern, 2005) than in non-musicians in several areas, including the precentral gyrus and the superior parietal cortex (See Figure 9).

**Figure 10:**
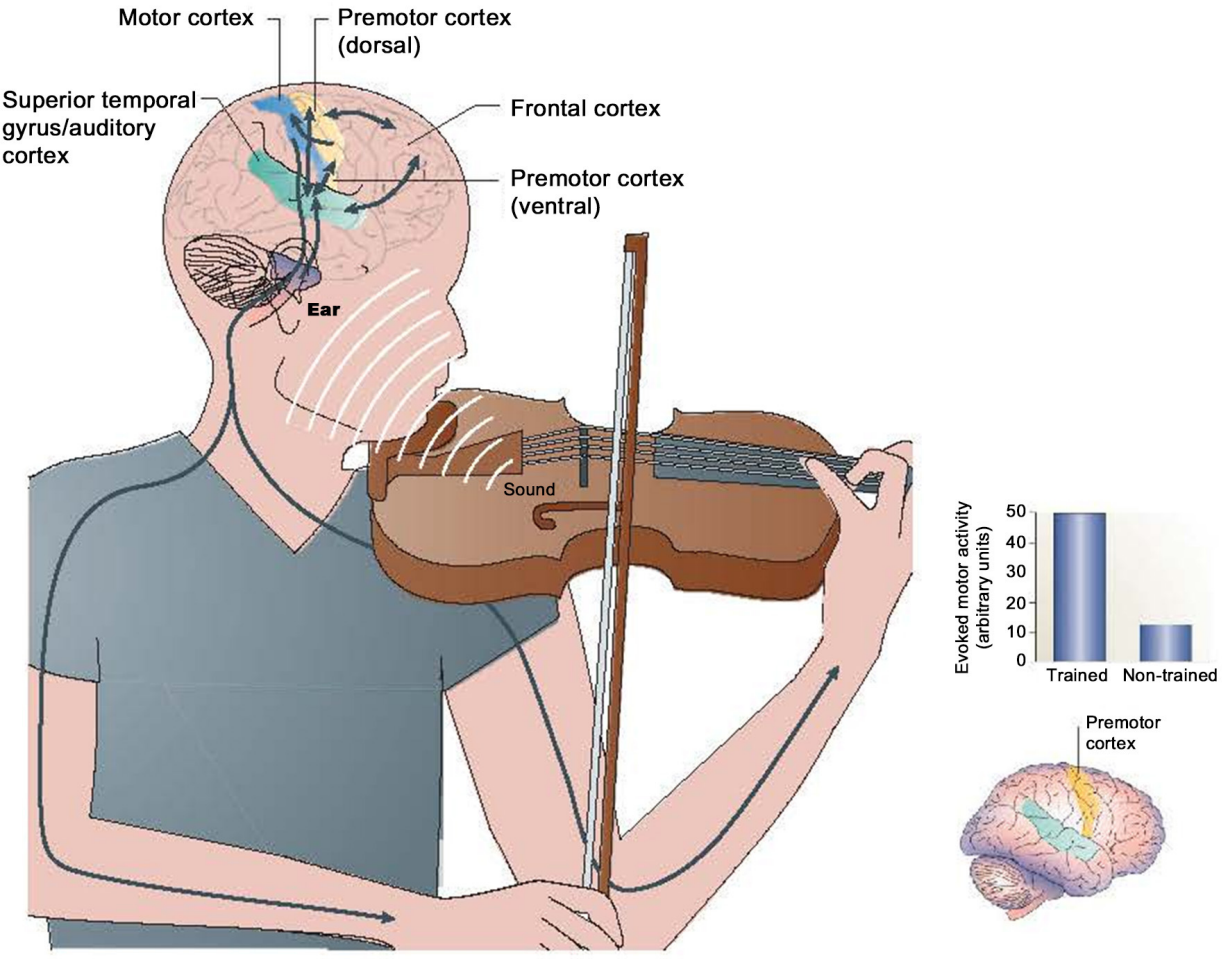
**(Left) Interaction of the auditory and motor systems during musical performance, and (right) associated premotor region changes in trained vs. non-trained musicians (Zatorre et al., 2007)**.

The premotor regions and the anticipatory cephalic organization of human action are linked throughout to musical expression. Neural action between premotor regions, auditory systems, and motor output are pervasive in musical expression and the organization of action (Zatorre, 2001; Patel, 2007; Koelsch, 2011). The dorsal premotor region in particular is knotted to metrical musical sensibilities (Zatorre, 2001). Regions of the premotor cortex may be particularly activated in experienced musicians during the execution of musical actions (See Figure 10) (Bangert et al., 2006; Baumann et al., 2007).

The neural correlates of musical exposure and practice indicate that music affects a broad array of human functioning, and that our cortex is built to receive music, process it, and change based on this exposure. These changes occur across neurological domains such that music affects pathways of audition, speech, language, memory, motor expression, and more. These neuronal changes demonstrate the importance of music to human functioning and how broadly it impacts our structural anatomy, as well as our behavior and social functioning in the world.

## Conclusions

Music makes clear that there is no mind-body separation. The rhythmicity of the brain, along with the development of cognitive capabilities, illustrates clear how inherent music is to our evolutionary and social success. This social link demonstrates that biological and cultural evolution are intertwined in music.

Based on this, we can predict that imagining music and listening to music would activate many of the same brain regions, which indeed it does. Additionally, music facilitates social contact and would therefore be linked to an expanding cortex, which indeed, cortical expansion it. We could further predict that music would contribute to social cooperative behaviors, and that genetic syndromes like Williams syndrome, with exaggerated social approach behaviors, would also reveal a greater propensity for music, a fundamental prosocial feature. Biologically, oxytocin, a prosocial facilitating peptide, may be elevated in Williams syndrome. Like dopamine, oxytocin may be elevated in listening to music.

Music emerged as part of communicative capability, a universal feature long noted and discussed (Juslin and Sloboda, 2001; Cross, 2009). Indeed Rousseau goes so far as to suggest “that the first language of the human race was song and many good musical people have hence imagined that man may well have learned that song from the birds (Rousseau, 1966, p.136).”

Like language, the roots of music may be in the inherent shared features of our social brain, allowing us to communicate with others. Since its development, music has filled many other important roles for humans.

Music is a fundamental part of our evolution; we probably sang before we spoke in syntactically guided sentences (Mithen, 1999, 2009; c.f. Pinker, 1994). Song is represented across animal worlds; birds and whales produce sounds, though not always melodic to our ears, but still rich in semantically communicative functions. Song is not surprisingly tied to a vast array of semiotics that pervade nature: calling attention to oneself, expanding oneself, selling oneself, deceiving others, reaching out to others, and calling on others. The creative capability so inherent in music is a unique human trait.

Ian Cross, a professor at the faculty of music at Cambridge University, has noted that facilitating the transmission of information across shared social intentional space is the pervasive social milieu; evolutionary factors are critical in understanding musical sensibility (Cross, 2009), specifying diverse social contexts in relationships. We use music because it expands our communicative social contact with one another. We also enjoy music even without obvious instrumental features. Music, like other features about us, became a worthy end for its own sake.

Music is about communication; our evolutionary ascent is the scaling of communicative competence, tracing constants of musical sensibilities to common points of origins of humanity and expansion of musical expression from this common source in prehistorical times (Grauer, 2006). But musical expression is about much more than that. Musical sensibility pervades our social space and our origins in synchrony with our interactions with others that are built on core biological propensities (Brown et al., 2004; Merker, 2005).

A series of steps set the condition for this core capability in our species. A change in the vocal apparatus, leading to a larynx of a certain size, shape, and flexibility, is but one example. A vocal capability tied to social awareness along with other cephalic capabilities, converged together in behavioral coherence.

The evolutionary record suggests that musical instruments were perhaps well expressed over 50,000 years ago in simple flutes and pipes (Cross, 1999; Morley, 2003) and were depicted in our art (e.g., on bison horn). What began as an extension of communication in a social context became something greater, which was enjoyed in itself. Our evolution is tightly bound to music and to the body as an instrument (e.g., clapping). Music, amongst other things, helps to facilitate social cooperative and coordinated behaviors (Brown, 2006).

Music permeates the brain as a core feature, from pitch and rhythm to tempo and affect (Patel, 2007). The melodies dance across our brain, memory guides them through our lives, and the tension and release, or resolution, form an outstanding aspect of the experience of many forms of music and neural processing of events (Steinbeis and Koelsch, 2007).

### Conflict of interest statement

The authors declare that the research was conducted in the absence of any commercial or financial relationships that could be construed as a potential conflict of interest.
